# Brazil towards malaria elimination: A time-series analysis of imported cases from 2007 to 2022

**DOI:** 10.1371/journal.pgph.0003822

**Published:** 2024-10-11

**Authors:** Klauss Kleydmann Sabino Garcia, Gabriel Z. Laporta, Seyi Soremekun, Christian Bottomley, Amanda Amaral Abrahão, Gilberto Gilmar Moresco, Chris Drakeley, Anielle de Pina Costa, André M. Siqueira

**Affiliations:** 1 Faculty of Health Sciences, University of Brasilia, Brasilia, Federal District, Brazil; 2 Nucleus of Tropical Medicine, University of Brasilia, Brasilia, Federal District, Brazil; 3 Graduate Program in Health Sciences, FMABC Medical School University Center, Santo André, São Paulo, Brazil; 4 Department of Infection Biology, London School of Hygiene & Tropical Medicine, London, United Kingdom; 5 MRC International Statistics and Epidemiology Group, London School of Hygiene & Tropical Medicine, London, United Kingdom; 6 Health and Environmental Surveillance Secretariat, Ministry of Health, Brasilia, Federal District, Brazil; 7 Fluminense Federal University, Niterói, Rio de Janeiro, Brazil; 8 Evandro Chagas National Institute of Infectious Diseases, Fundação Oswaldo Cruz (Fiocruz), Rio de Janeiro, Rio de Janeiro, Brazil; Yale School of Public Health, UNITED STATES OF AMERICA

## Abstract

Malaria is a global health challenge, and international efforts are underway to alleviate its impact by 2035. Within the 249 million global cases, 0.6 million occur in the Americas, mainly in Venezuela, Brazil, and Colombia. Considering Brazil’s geographical proximity to malaria-endemic countries in South America, this study objective is to analyze the epidemiological characteristics and time trends of imported malaria cases in Brazil from 2007 to 2022, discussing their influence on the elimination process. This is an ecological time-series study that analyses malaria imported cases (infected in other countries) notified in Brazil, from 2007 to 2022. Brazil’s Ministry of Health data were used. Descriptive statistics were employed to analyze sociodemographic and spatial patterns, while the impact of the Covid-19 pandemic on imported malaria trends was assessed using Prais-Winsten regression methods. In the study period there was a total of 109,914 imported cases (2.6% of Brazil’s total malaria burden). There was an annual reduction of 515.3 cases (p = 0.001) prior to the Covid-19 pandemic. During the pandemics there was an overall reduction of -3,301.8 cases (p = 0.001). In the Amazon region *P*. *vivax* imported infections predominated, whereas in the extra-Amazon region *P*. *falciparum* imported infections were more prevalent. Most imported cases were males (67.8%), of Black ethnicity (47.5%), with incomplete primary education (45.1%), aged 20–39 (61.1%), and primarily gold miners (54.0%). Most cases are from French Guiana (31.7%), Venezuela (30.0%), and Guyana (17.9%). African nations, notably Angola and Nigeria, were primary sources of imported cases to the extra-Amazon region. The imported cases flux, predominantly from Latin America, threatens Brazil’s elimination goals by potentially reintroducing the disease into previously cleared areas and sustaining the transmission in endemic areas. Strengthening epidemiological surveillance at the borders and fostering international cooperation are imperative steps in addressing this challenge.

## Introduction

The global landscape of infectious diseases is marked by the menacing presence of malaria, a highly burdensome tropical disease afflicting mainly the African region, the Eastern Mediterranean Region, the South-East Asia Region, the Western Pacific Region and the Americas region [1]. The heightened prevalence of this disease in these regions is inextricably linked factors such as clime, local geography, weakened economic situation and environments conducive to disease transmission. Recognizing the urgency of the situation, global initiatives have set their sights on the ambitious goal of controlling and ultimately eliminating malaria by the year 2035 [2, 3].

Malaria is caused by the Plasmodium parasite transmitted through *Anopheles* mosquito bites [4]. There are five species of malaria that affect humans: *Plasmodium vivax*, *Plasmodium falciparum*, *Plasmodium ovale*, *Plasmodium malariae*, *and Plasmodium knowlesi* [2]. Of particular concern are severe malaria cases, often associated with *P*. *falciparum* infections or mixed infections [5]. In Africa, *falciparum* malaria is predominant, while South America grapples with the prevalence of *vivax* malaria [6].

In 2022, malaria continued to cast a daunting shadow over the global health landscape. A staggering 249 million cases were reported worldwide, with an awful 94% of those cases concentrated in the African region, and around 600,000 cases in South America [1]. Within South America, malaria’s prevalence varies significantly among countries, with the Amazon region emerging as a notable focus of transmission. According to the World Health Organization (WHO), Venezuela, Brazil, and Colombia are the primary culprits, jointly accounting for about 79% of all cases in the Americas [1].

In Brazil, malaria’s local transmission mainly occurs in the Amazon region, with about 99% of cases emanating from this area. The non-endemic extra-Amazon region is spared from endemic transmission [7–9]. The porous borders that Brazil shares with economically fragile neighboring countries play a pivotal role in fueling local transmission, making imported malaria cases a significant concern [10].

The occurrence of imported malaria cases is intricately connected to factors such as international travel, migration, and commercial or economic activities [10, 11]. These activities inadvertently facilitate the dissemination of parasites and vectors, posing a formidable challenge for disease control efforts. Swift diagnosis and treatment responses are crucial in curtailing the spread of the disease, this necessitates a more profound understanding of the epidemiological implications and prevention strategies [10].

The WHO definitions regarding the classification of malaria cases, distinguishing between autochthonous and imported cases, play a pivotal role in disease identification and control [12, 13]. Autochthonous cases denote locally transmitted infections, while imported cases involve individuals who contracted the disease in endemic regions and introduced it to non-endemic areas [12].

In this complex and pressing context, this study aims to analyze the epidemiological characteristics and time trends of imported malaria cases in Brazil from 2007 to 2022. This endeavor seeks to shed light on the dynamic epidemiological scenario while discussing topics that contribute to fulfilling Brazil’s malaria elimination goals.

## Methods

### Study design, period, and study population

An informative ecological study was carried out, focusing on a time series analysis of imported malaria cases in Brazil. The study’s timeframe extended from 2007 to 2022. The initial year, 2007, was chosen to align with the implementation of the Sinan System in the extra-Amazon region. Although the Sivep-Malaria system was implemented in 2003, starting the analysis in 2007 ensures a standardized timeframe. To assess the potential impact of the Covid-19 pandemic on the reporting of imported cases in Brazil, it was assumed that the pandemic commenced following the initial global cases in December 2019, thus encompassing the entire years of 2020, 2021, and 2022.

The study population comprised all reported cases of imported malaria in Brazil, according to the WHO’s case definition, which identifies imported malaria cases as those originating from recognized malaria-endemic areas outside the country where the diagnosis was made [12]. Only imported cases with laboratory confirmation were included, ensuring data accuracy and reliability. Additionally, records were considered if the "Country of Infection" field was completed, except for entries labeled as “unknown" or left blank. This approach was employed to maximize the quality and relevance of the data under examination.

### Database sources

De-identified data from positive malaria cases reported in the Information System for Notifiable Diseases (Sinan) from the Ministry of Health of Brazil (BMoH) were utilized, capturing notifications related to cases recorded in the extra-Amazon region of Brazil. Access to these data was facilitated through the TABNET/DATAUS portal [14]. Additionally, data from the Malaria Epidemiological Surveillance System (Sivep-Malaria), which receives notifications from the Amazon region, were also incorporated. The latter dataset was made available by the BMoH under protocol #25072.032117/2023-34.

### Variables used

The following variables were utilized in the study: Notification date; Detection type; Patient’s age; Gender; Race/ethnicity; Education level; Occupation/activities in the last 15 days; Country of infection; Reporting State (local where the case was detected); Laboratory test result for *Plasmodium falciparum*, *Plasmodium vivax* or mixed infection (*Pf* + *Pv* or *Pf* + *Po*); and Parasitemia.

### Ethical aspects, data availability and software used

This study did not necessitate authorization from research ethics committees, as it exclusively relied on anonymized data. The data were freely available and provided by the BMoH, in accordance with guidelines established in resolution No. 466/202 of the National Health Council.

The data used in this study are available in S1 Data.

### Statistical analysis

For data reading, data processing, and time series analyses, the R software (version 4.2.1) [15] was utilized. Missing data levels were quantified and included in the descriptive profile analysis. Additionally, to select malaria cases infected in other countries, only notifications with the "country of infection" field correctly filled out were included.

### Trend analysis

For trend analysis, a regression model was utilized to ascertain any discernible increase or decrease in the temporal trend of imported malaria cases. The regression model incorporated "malaria cases per year” on the log_10_ scale as the dependent variable, with years serving as a continuous independent variable. To mitigate the issue of temporal autocorrelation between consecutive residuals, the Prais-Winsten method was used [16]. The trend was expressed as [17], a relative percentage change per year, by back transforming the slope coefficient (β) obtained from linear regression model [18]:

$$Annual\;Percent\;Change=(-1+10^{\left(\beta \right)})\times 100$$

$$Confidence\;interval\;95\%=(-1+10^{\left(\beta \;\pm \;t\;value\;*\;Standard\;error\right)})\times 100$$

### Impact analysis of the pandemic

For the impact analysis, we adopted the interrupted time series methodology [19, 20]. This involved applying the same model as described earlier for trend analysis, but with the addition of an extra covariate to represent the effect of the pandemic. The pandemic variable was set to ’0’ for years before the pandemic and ’1’ for years following its onset [21, 22]. This model, as per Antunes and Cardoso [18], interprets the effect of the pandemic as a change in level or step. As with the trend analysis, autocorrelation was addressed using the Prais-Winsten method [20].

### Spatial analysis

The Qgis software (version 2.18) [23] was used to plot data of the flux analysis. For the flux analysis the plugin “Flowmaps” was used. The flux analysis considers the total number of cases reported in foreign countries and the states where they were diagnosed was tallied, allowing for the creation of a matrix indicating the "origin" and "destination" of infection.

## Results

**Fig 1:**
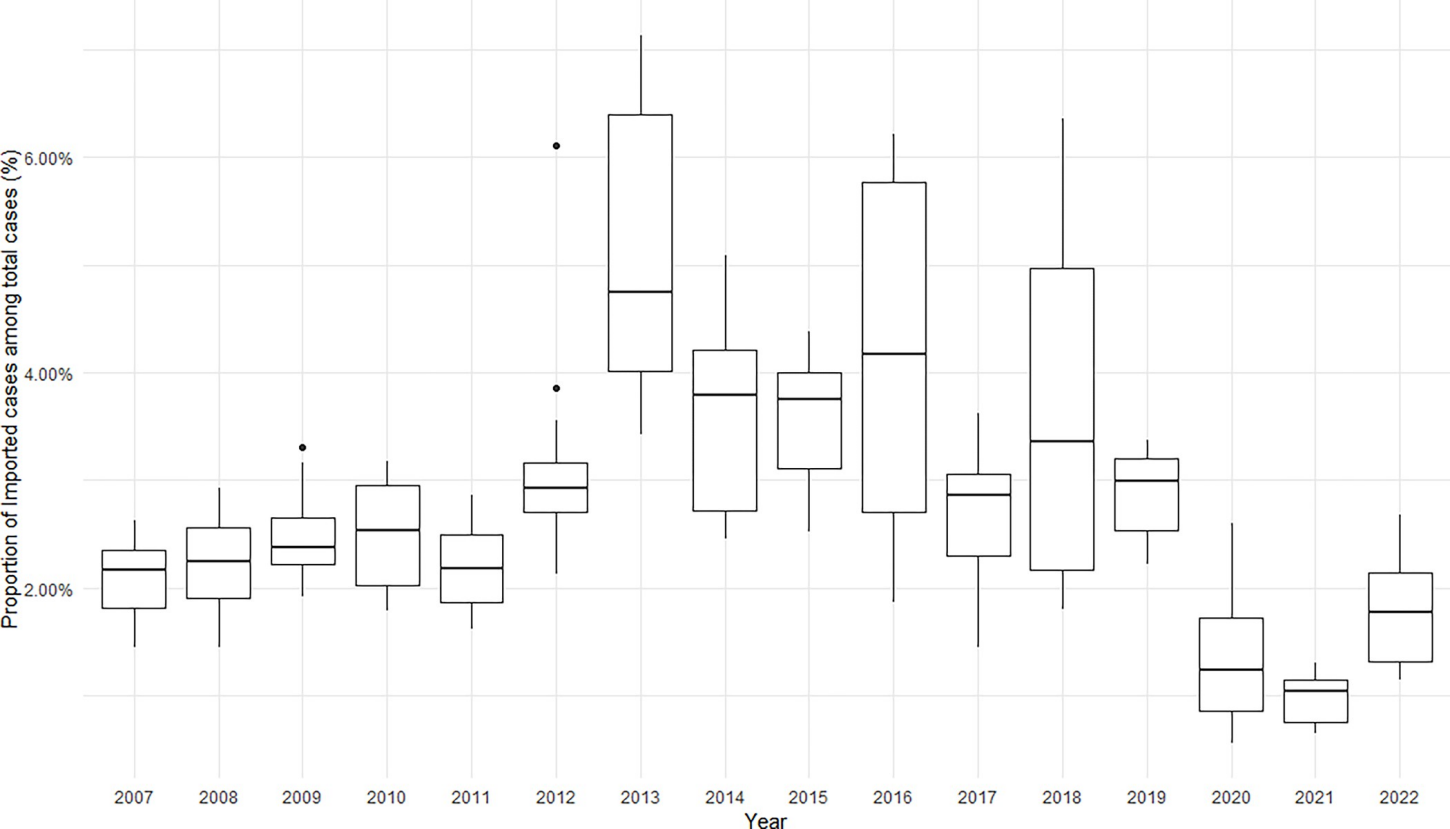
Proportion of imported malaria cases in Brazil from 2007 to 2022.

During the analyzed period, a total of 109,914 cases of imported malaria were reported in Brazil. This accounts for 2.6% of the total reported cases in Brazil (autochthonous and imported combined) which totaled 4,176,982 between 2007 and 2022. Historically, the proportion of imported cases in Brazil has remained below 5%. Prior to 2020, the average proportion of imported cases ranged between 2% and 5%. In 2020, amidst the pandemic, this proportion dropped to approximately 1%. By 2022, the average proportion of cases exhibited a modest increase, nearing 2% (see Fig 1).

Among the 109,914 imported cases, 3,455 were reported in the extra-Amazon region, representing 3.1% of the total imported cases. Conversely, the Amazon region accounted for most cases, with 106,459 reported infections, constituting about 96.9% of the total imported cases.

**Fig 2:**
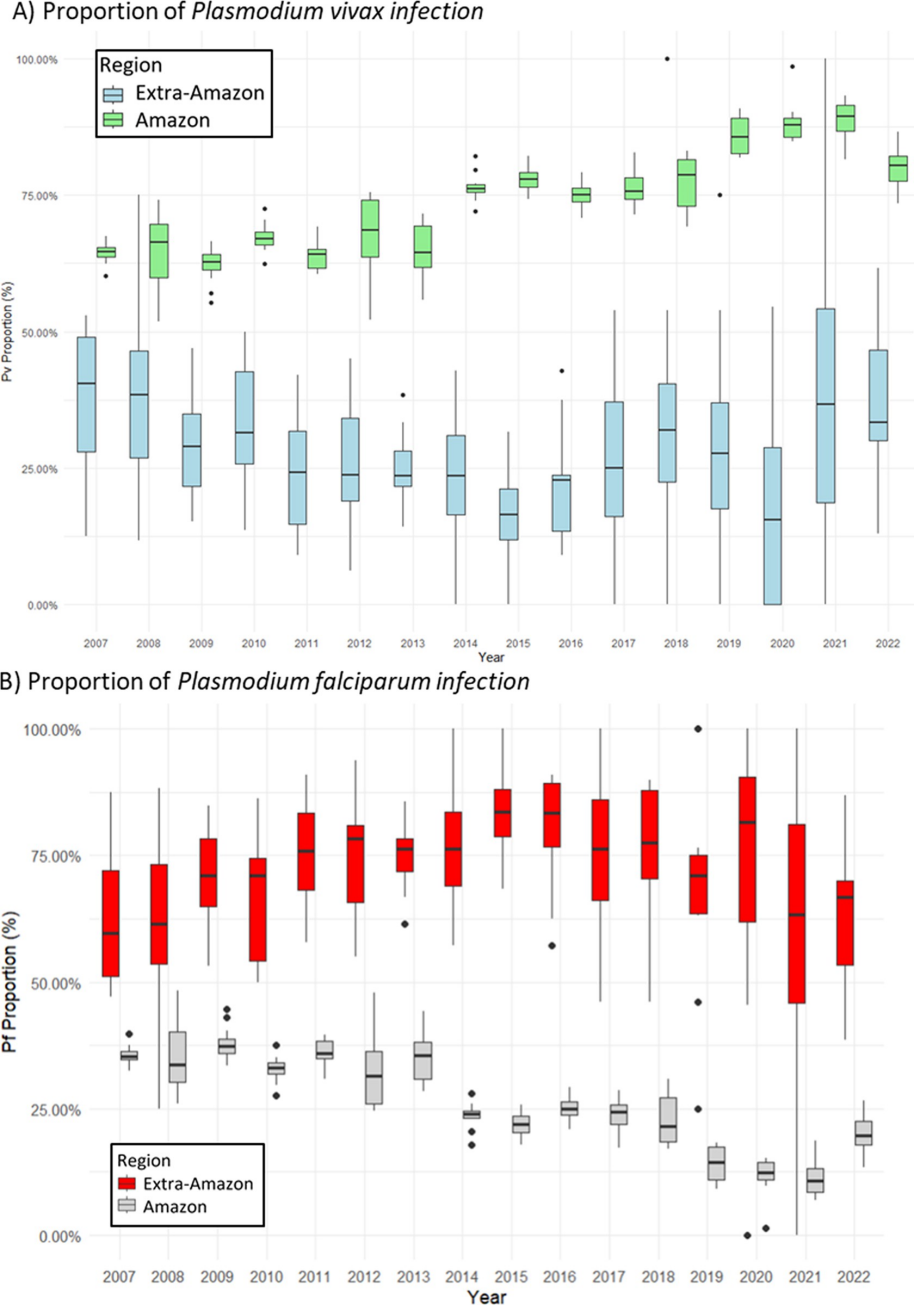
Proportion of imported malaria cases by species, Brazil, 2007–2022.

The predominant imported cases reported in the Amazon region are *P*. *vivax* infections, with an average proportion of 73.8% observed throughout the analyzed period, as depicted in Fig 2A. In contrast, in the extra-Amazon region, the proportion of imported *P*. *falciparum* infections demonstrates fluctuations around a mean of 71.4%, as illustrated in Fig 2B.

**Fig 3:**
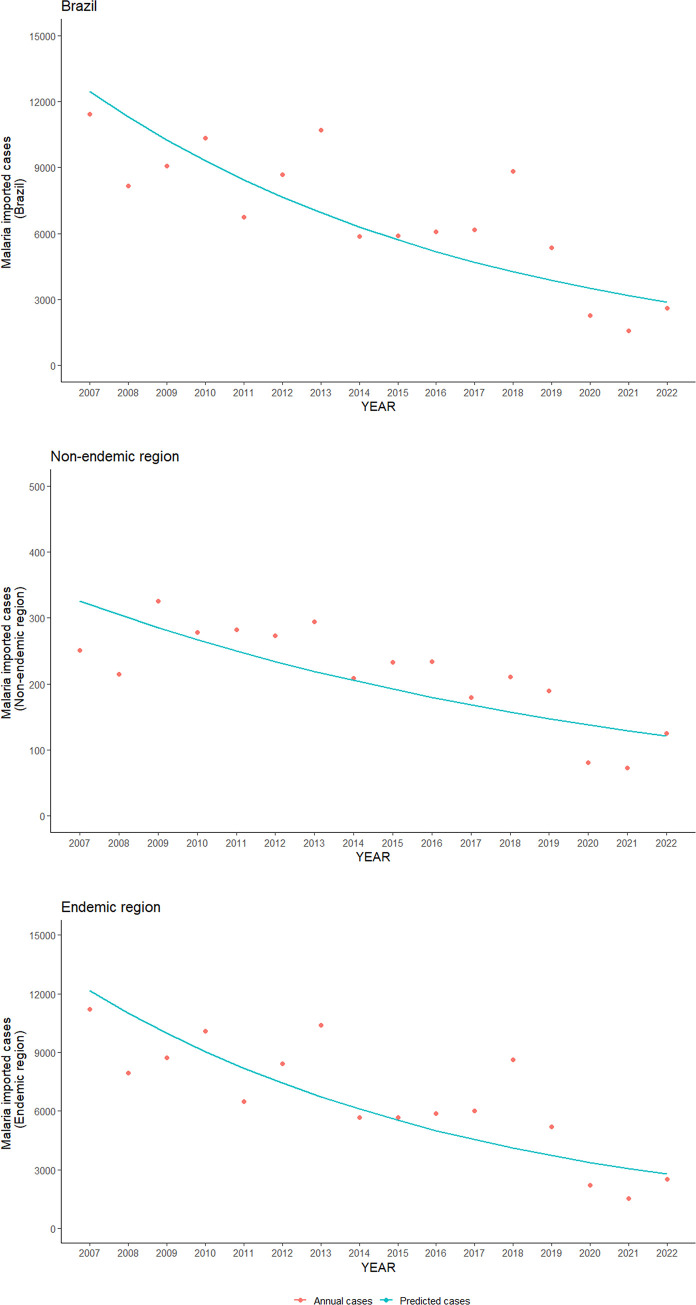
Temporal trends of imported malaria cases in Brazil between 2007–2022.

The trend analysis estimates an absolute decreasing trend of -515.3 imported malaria cases per year in the period (-731.5; -299.1, p-value: 0.001). Specifically, in the Amazon region, the trend was -502.9 per year (-714.9; -291.0, p-value: 0.002), and in the extra-Amazon region, it was -11.8 per year (-18.2; -5.5, p-value: 0.007) (Fig 3).

The Annual Percent Change of imported malaria cases in Brazil was -20.1% (CI: -29.1%; -9.9%; p-value: 0.002). In the Amazon region, it was -20.3% (CI: -29.4%; -9.9%; p-value: 0.002), while in the Extra-Amazon region, it stood at -14.1% (CI: -21.8%; -5.6%; p-value: 0.007).

**Fig 4:**
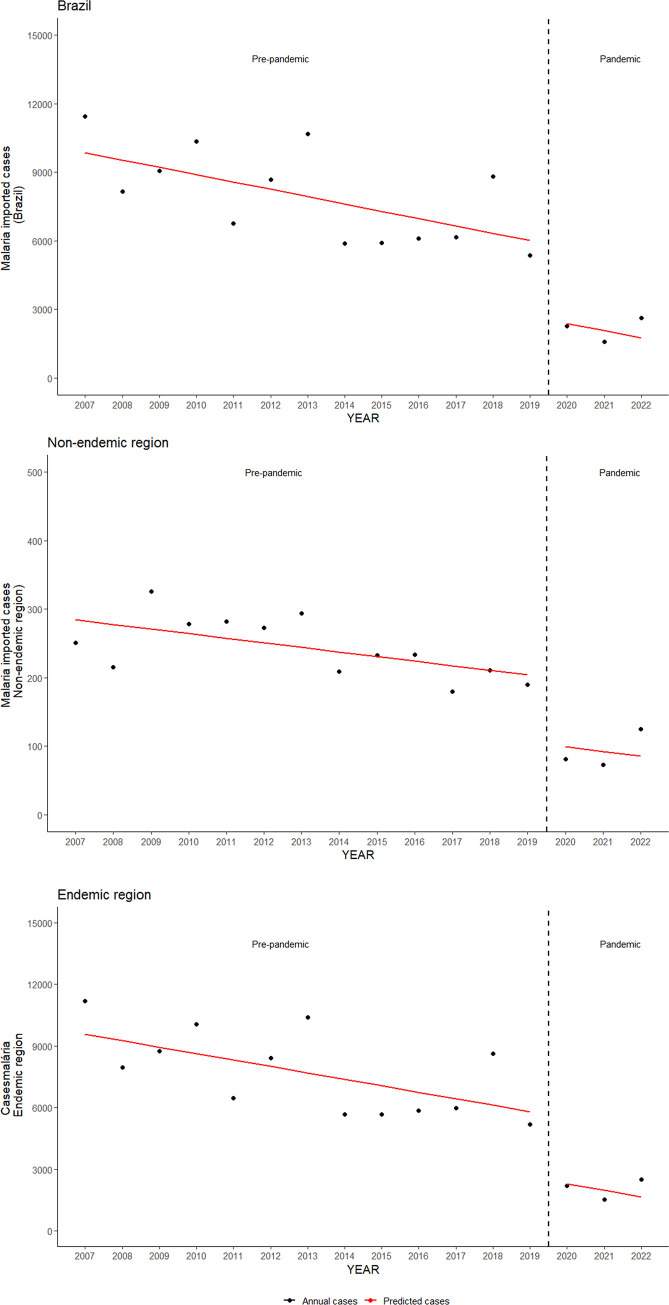
Impact of the pandemic on the occurrence of imported malaria cases in Brazil.

It’s evident from Fig 3 that starting from 2020, the total number of imported cases was lower than the predicted total in the regression model. To estimate the effect of the Covid-19 pandemic in 2020 and beyond, the interrupted time series analysis demonstrated a statistically significant overall reduction in the number of reported malaria cases in Brazil by -3,301.8 (95% CI: -5,474.4; -1,129.1; p-value: 0.01) cases in the post-pandemic period. In the Amazon region, this reduction was -3,201.0 (-5,363.3; -1,038.7, p-value: 0.01), and in the extra-Amazon region, it was -98.2 (-157.6; -38.8, p-value: 0.006) (Fig 4).

The epidemiological profile of the period highlights that imported cases were predominantly in males (N = 74,544; 67.8%), individuals of black race/ethnicity (N = 52,174; 47.5%), with incomplete primary education (N = 49,626; 45.1%), aged between 20 and 39 years (N = 67,121; 61.1%), and more than half were gold miners (N = 59,375; 54.0%). Most cases were reported through passive detection (N = 92,724; 84.4%), and *P*. *vivax* infections predominated (N = 75,731; 68.9%). The most prevalent parasitemia was recorded as up to 10,000 parasites per mm^3^ (N = 99,566; 90.6%) (Table 1). Interestingly, the extra-Amazon region epidemiological profile differ from the Amazon region in some aspects.

**Table 1:** Epidemiological profile of imported malaria cases reported in Brazil, 2007–2022.

	Notified in the	%	Notified in the	%	Total	%
	Extra-Amazon region		Amazon Region		Imported Cases	
Total	3,455	3.1	106,459	96.9	109,914	100.0
**Age (Mean and SE)**	35.8 (13.4)		30.1 (13.2)		30.3 (13.3)	
**Sex**						
Male	2,766	80.1	71,778	67.4	74,544	67.8
Female	688	19.9	34,675	32.6	35,363	32.2
Unknown/Blank	1	0.0	6	0.0	7	0.0
**Race/Ethnicity**						
Black	1,840	5.3	51,990	48.8	52,174	47.5
White	1,173	34.0	6,895	6.5	8,068	7.3
Yellow	46	1.3	1,084	1.0	1,130	1.0
Indigenous	32	0.9	4,005	3.8	4,037	3.7
Unknown/Blank	364	10.5	42,485	39.9	42,849	39.0
**Education**						
Illiterate	31	0.9	7,191	6.8	7,222	6.6
Incomplete Elementary School	452	13.1	49,174	46.2	49,626	45.1
Complete Elementary School	204	5.9	12,817	12.0	13,021	11.8
Incomplete High School	207	6.0	7,043	6.6	7,250	6.6
Complete High School	509	14.7	9,639	9.1	10,148	9.2
Higher Education	759	22.0	3,224	3.0	3,983	3.6
Not Applicable	93	2.7	4,303	4.0	4,396	4.0
Unknown/Blank	1,200	34.7	13,068	12.3	14,268	13.0
**Age Group**						
<1	31	0.9	710	0.7	741	0.7
01–04	47	1.4	3,060	2.9	3,107	2.8
05–09	31	0.9	2,978	2.8	3,009	2.7
10–19	152	4.4	11,381	10.7	11,533	10.5
20–39	1,913	55.4	65,208	61.3	67,121	61.1
40–59	1,139	33.0	21,264	20.0	22,403	20.4
60 or Older	142	4.1	1,858	1.7	2,000	1.8
Unknown/Blank						
**Occupation/Activity in Last 15 Days**						
Mining/gold mining	474	13.8	58,901	55.3	59,375	54.0
Agriculture and Fishing	191	5.5	11,579	10.9	11,770	10.7
Domestic Work	56	1.6	4,042	3.8	4,098	3.7
Road/ Dam Construction	221	6.4	171	0.2	392	0.4
Traveler/Turism	1,184	34.3	2,795	2.6	3,979	3.6
Other	1,023	29.6	18,492	17.4	19,515	17.8
Unknown/Blank	306	8.9	10,479	9.8	10,785	9.8
**Detection Type**						
Passive Detection	2,284	66.1	90,440	85.0	92,724	84.4
Active Detection	912	26.4	7,374	6.9	8,286	7.5
Unknown/Blank	259	7.5	8,645	8.1	8,904	8.1
**Parasitic Species (Lab Result)**						
*P*. *vivax*	928	26.9	74,803	70.3	75,731	68.9
*P*. *falciparum*	2,271	65.7	27,377	25.7	29,648	27.0
Mixed infection	178	5.2	4,178	3.9	4,356	4.0
*P*. malariae	27	0.8	99	0.1	126	0.1
*P*. *ovale*	51	1.5	2	0.0	53	0.0
Unknown/Blank						
**Parasitemia (mm** ^**3**^ **)**						
< 500 mm^3^	1,474	42.7	44,597	41.9	46,071	41.9
501–10,000 mm^3^	1,039	30.1	52,456	49.3	53,495	48.7
10,001–100,000 mm^3^	657	19.0	7,313	6.9	7,970	7.3
>100,000 mm^3^	285	8.2	298	0.3	583	0.5
Unknown/Blank			1,795	1.7	1,795	1.6
**Probable Infection Region**						
South America	945	27.4	105,847	99.4	106,792	97.2
Africa	2,362	68.4	217	0.2	2,579	2.3
North, Central, and Caribbean America	61	1.8	16	0.0	77	0.1
Asia and Oceania	69	2.0	18	0.0	87	0.1
Europe	18	0.5	361	0.3	379	0.3
Unknown/Blank						

S.E.: Standard error. Source: Sivep-Malaria, Sinan—Ministry of Health of Brazil. Data updated in June 2023.

In the extra-Amazon region malaria cases are more frequent among individuals of white skin color (34.0%) and with Higher education (22.0%), while in the Amazon region cases are more frequent among black skin color individuals (48.8%) and ones with incomplete elementary school (46.2%). Also, the proportion of cases with more than 10,000 parasites per mm^3^ is higher than in the Amazon region, due to the higher proportion of *P*. *falciparum* cases in the extra-Amazon region (Table 1).

The regions that contribute with most cases to Brazil were South America (N = 106,792; 97.2%) and Africa (N = 2,579; 2.3%). However, the cases detected in the Amazon region were predominantly from South America, comprising 99.4% of the total. Among the imported cases from Africa, the majority (91.6%) were reported in the extra-Amazon region. Conversely, among the imported cases from the extra-Amazon region, cases originating from the African continent constitute 68.4% (Table 1).

Among the countries exporting the highest number of cases to Brazil, 98.4% of cases were concentrated in nine countries: French Guiana (N = 34,830; 31.7%), Venezuela (N = 32,980; 30.0%), Guyana (N = 19,657; 17.9%), Peru (N = 12,063; 11.0%), Bolivia (N = 4,539; 4.1%), Suriname (N = 2,118; 1.9%), Angola (N = 1,113; 1.0%), Colombia (N = 518; 0.5%), South Africa (N = 369; 0.3%). The first six listed were the main exporters to the Amazon region. The primary exporters to the extra-Amazon region include Angola (N = 1,031 cases; 30.1%), French Guiana (N = 337; 9.8%), South Africa (N = 298; 8.6%), Nigeria (N = 250; 7.2%), Guyana (N = 211; 6.1%), Mozambique (N = 210; 6.1%), Venezuela (N = 204; 5.9%), and Equatorial Guinea (N = 103; 3.0%).

In 2022, the top four countries with the highest number of cases exported to Brazil were: Venezuela (N = 658; 25.1% of 2.620 imported cases in 2022), Peru (N = 730; 27.9%), Guiana (N = 642; 24.5%) and Bolivia (N = 264; 10.1%). In contrast, French Guyana was responsible for only 190 imported cases (7,2%).

The highest parasitemia (above 10,000 parasites/mm^3^) came from the countries Venezuela (3,054; 35.7%), French Guiana (2,085; 24.4%) and Guyana (1,628; 19.0%). Among the countries on the African continent, Angola (347; 4.1%), South Africa (118; 1.4%), Mozambique (92; 1.1%) and Nigeria (91; 1.1%) stood out.

Among the states in Brazil that received the most cases with parasitemia (more than 10,000 parasites/mm^3^) were Roraima (4,204; 49.2%), Amapá (1,387; 16.2%) and Amazonas (733; 8.6%), all of which are border regions.

**Fig 5:**
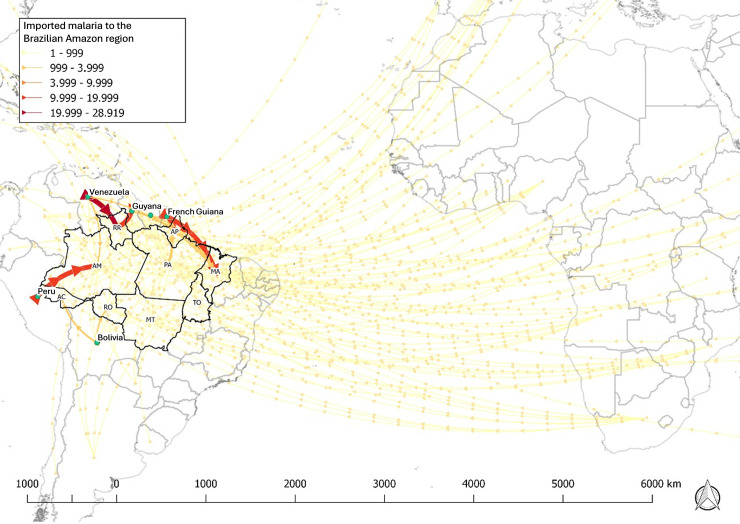
International flux of malaria imported cases to endemic Amazonian area in Brazil between 2007–2022. Shapefiles were downloaded using from the Brazilian Institute of Geography and Statistics (IBGE): (url: https://www.ibge.gov.br/geociencias/downloads-geociencias.html; Terms of use: https://geoftp.ibge.gov.br/organizacao_do_territorio/malhas_territoriais/malhas_municipais/municipio_2022/Leia_me.pdf); World countries shapefiles were downloaded from the ArcGIS Hub (url: https://hub.arcgis.com/datasets/esri::world-countries-generalized/explore; Terms of use: https://goto.arcgis.com/termsofuse/viewtermsofuse).

**Fig 6:**
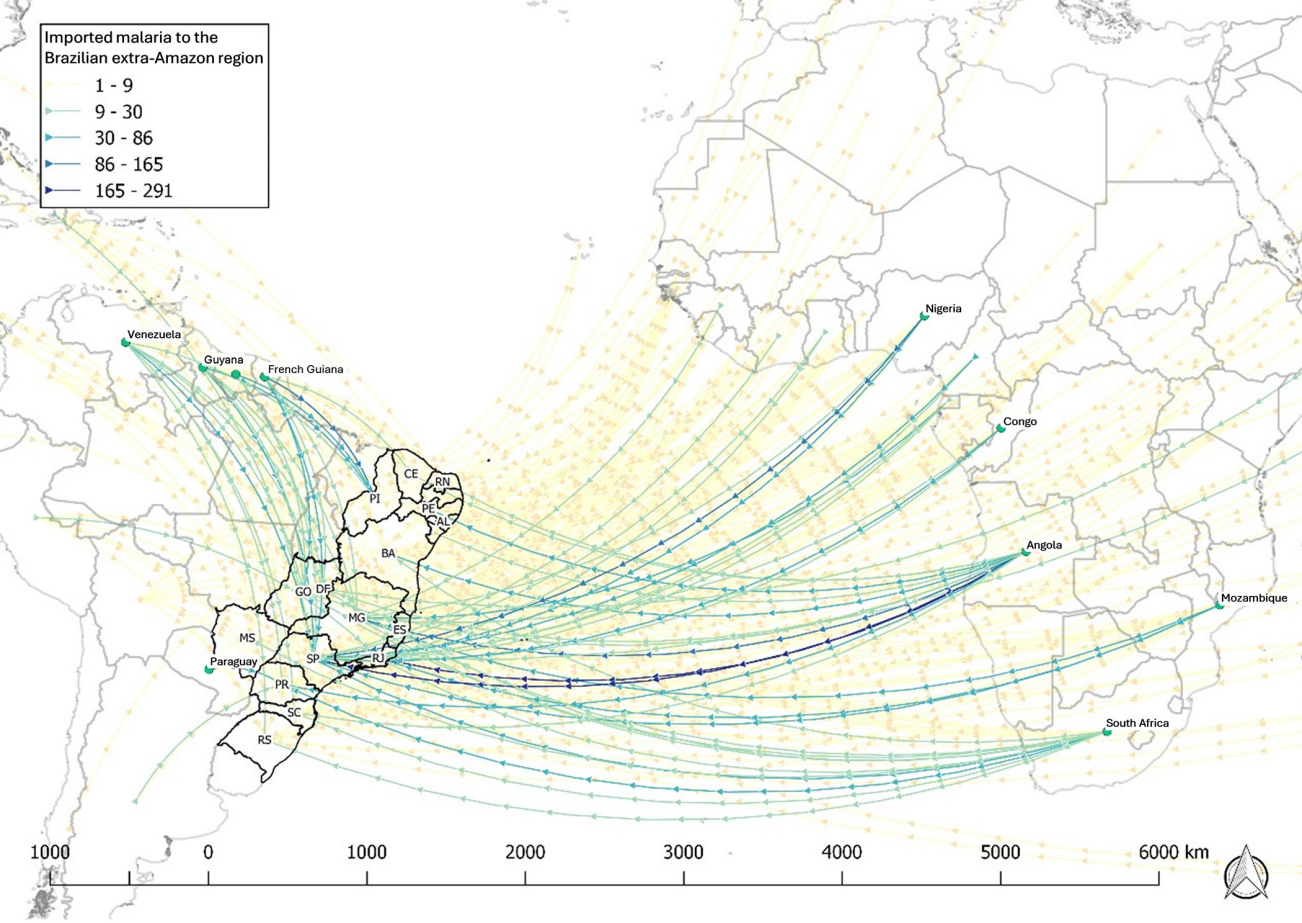
International flux of malaria imported cases to non-endemic extra-Amazonian region in Brazil between 2007–2022. Shapefiles were downloaded using from the Brazilian Institute of Geography and Statistics (IBGE): (url: https://www.ibge.gov.br/geociencias/downloads-geociencias.html; Terms of use: https://geoftp.ibge.gov.br/organizacao_do_territorio/malhas_territoriais/malhas_municipais/municipio_2022/Leia_me.pdf); World countries shapefiles were downloaded from the ArcGIS Hub (url: https://hub.arcgis.com/datasets/esri::world-countries-generalized/explore; Terms of use: https://goto.arcgis.com/termsofuse/viewtermsofuse).

As shown in Fig 5, the importation flux was more intense between the neighboring countries of the Brazilian Amazon region. The most intense flux happened from Venezuela to Roraima, from Guyana to Roraima, from French Guiana to Maranhão, and from Peru to Amazonas. In the extra-Amazon region, the most intense flux of imported cases came from African countries, primarily from Angola to Rio de Janeiro and São Paulo, and from Nigeria to São Paulo (Fig 6).

Additionally, African imported cases accounted for 2,579 cases, which were predominantly *P*. *falciparum* infections (84.1%). The proportion of *P*. *vivax* infections imported from the African continent varied between 3.2% and 24.1% during the study period.

In Brazil, 92.0% of the imported cases were reported in 5 states: Roraima (N = 44,520; 40.5%), Amapá (N = 19,027; 17.3%), Maranhão (N = 16,277; 14.8%), Amazonas (N = 14,863; 13.5%), and Pará (N = 6,391; 5.8%). Specifically, in the extra-Amazon region, the states that reported the most imported cases were São Paulo (N = 1,018; 29.5%), Rio de Janeiro (N = 541; 15.7%), Minas Gerais (N = 369; 10.7%), Piauí (N = 316; 9.1%), and Paraná (N = 206; 6.0%). These states were responsible for more than 70.9% of the total cases registered in the extra-Amazon region.

## Discussion

### Main findings and comments

This study highlights that despite the historical number of imported cases of malaria in Brazil being approximately 2.6% in relation to the total number of cases reported in the country, these cases have considerable epidemiological relevance amid plans and actions that focus on the elimination of malaria in Brazil.

The analyses highlighted that there are two predominant profiles of these imported cases, the imported cases detected in the Brazilian Amazon region (endemic), which are mostly infected by *P*. *vivax* and originating from Latin American countries; the second profile consists of imported cases detected in the Brazilian extra-Amazonian region (non-endemic) that are mostly infected by *P*. *falciparum* and come from the African continent.

It was also highlighted that the epidemiological profile of these cases in the country is greatly influenced by cases that are reported in the Amazon region, which reports approximately 96.9% of all cases in the country. Thus, the sociodemographic profile was predominantly male, black, with low education, between 20 and 39 years of age and carrying out activities related to mining. The extra-Amazonian region presents a sociodemographic profile which is different from that of the Amazon region, with a higher occurrence of imported cases among white people, with higher education and a lower frequency of mining activities.

Furthermore, it was also found that the occurrence of imported cases in Brazil has shown a downward trend throughout the study period, but with a greater decline from 2020 onwards, as a likely effect of factors related to the Covid-19 pandemic, which began in December 2019, such as travel restrictions [24].

### Dangers associated with overseas Infections

While most of the African imported cases occurring in Brazil stem from *P*. *falciparum* infections, a notable 8.9% proportion of cases involve *P*. *vivax* infections from the same continent. This underscores the importance of monitoring *P*. *vivax* infections in Africa due to the risk of reintroducing this species into susceptible areas outside the extra-Amazon region [25].

It is important to acknowledge that acquiring malaria in endemic countries and traveling to non-endemic regions [25] such as the Brazilian extra-Amazon is a significant risk factor for developing severe malaria [26, 27]. The extra-Amazon presents a malaria lethality rate approximately 120 times higher than in the Brazilian Amazon [28]. In simpler terms, if you contract malaria outside an endemic country and be detected in the Brazilian extra-Amazon region in Brazil, you are at greater risk of experiencing severe malaria symptoms. This is due to the unusual custom of suspecting malaria in febrile cases, especially in regions affected by other febrile infectious diseases such as dengue. The delay in suspecting malaria, and the consequent delay in diagnosing and starting treatment, are determining factors in the process of developing severe malaria [25].

### Challenges to achieving elimination goals

Considering that Brazil aims to eliminate malaria by 2035 [29], the country should be aware of possible increases of imported cases in the following years and not to underestimate its occurrence, because the decrease due to the pandemics influence can provide the impression that imported cases are reducing, which is not true.

According to data from the International Migration Observatory of Brazil, migration from South American countries was on the rise until 2019 –exceeding 120,000 registrations of migrants to Brazil–falling to less than 60,000 migrants during the year 2020 and returning to rise in the following years. In 2023 it is estimated that there will be around 160,000 migrants in the country [30].

The movement of populations within the Latin American region significantly influences the incidence of imported malaria cases in Brazil. Many migrants arrive in Brazil seeking better living conditions and opportunities for economic development [31–33]. Consequently, the reduction in the number of imported malaria cases in Brazil post-2018 correlates with the overall decline of malaria within the Americas region [1]. Despite a general decrease in malaria cases in the Americas Region, Venezuela continues to report the highest incidence in the Americas [1]. Additionally, Venezuela is the country that contributes the most to migration patterns in Brazil [30].

The convergence of several factors in 2022—notably, the high malaria incidence in Venezuela, increased Venezuelan migration to Brazil post-pandemic, and the climatic effects associated with El Niño [34–36], suggests a potential rise in imported malaria cases.

That increase poses a risk to Brazil’s malaria elimination goals, as it could facilitate further transmission within the country. This risk pertains to the potential for imported cases to serve as reservoirs for malaria, reintroducing the parasite into regions where local transmission was previously eliminated [25]. Also, this resurgence can occur due to various factors, including the conducive environment of the Amazon region—characterized by dense rainforests and often suboptimal healthcare infrastructure [37]. A statistical modelling study could be conducted to simulate and assess the risk of malaria reintroduction in these receptive extra-Amazon areas.

Meanwhile, the extra-Amazon regions may exhibit lower immunity levels among local populations due to historically lower exposure to malaria, which could amplify outbreaks stemming from imported cases in receptive areas. Finally, there is a risk associated with the relaxation of surveillance and control measures, as areas previously declared malaria-free might reduce their vigilance following temporary elimination [37, 38].

### Obstacles in managing imported cases

One of the challenges for controlling imported cases is to do so inside mining areas. Health surveillance teams often work in isolation to combat diseases in areas where mining activities are predominantly clandestine. It is recognized that the healthcare sector primarily shoulders the responsibility for addressing this issue, despite it extending beyond the healthcare system [39].

At Brazil’s border with neighboring countries, the northern region of Brazil experiences a high occurrence of imported cases [38, 40]. Historically, these activities have been related to the migration of vulnerable populations and the presence of mining activities. It is worth noting that mining activities lead to the flux of workers between Brazil and neighboring countries [32, 41]. Furthermore, the occurrence of deforestation and mining activities itself is associated with an increase in *P*. *falciparum* cases [42, 43]. Therefore, the occurrence of this type of activity can increase imported cases of *P*. *falciparum* and trigger outbreaks in areas where this species is not endemic.

Strengthening malaria epidemiological surveillance in frontiers is not going to be enough to cease the flux of infected individuals. Engaging additional societal and governmental sectors is essential to address the intricate issues of population vulnerability and illegal mining activities, which are linked to high rates of malaria transmission [40]. For that, Brazil’s government must work alongside with the Pan American Health Organization (PAHO) to fortify cooperation among South American countries to help control the flux of infected cases between countries.

Despite Brazil’s efforts to eliminate malaria deaths by 2030 and autochthonous cases by 2035, imported cases will persist in the country. Similar situations occur in countries like China and Australia [44, 45]. Consequently, the risk of reintroduction and the emergence of severe forms of the disease remain, particularly in the extra-Amazonian region. Enhancing detection speed and strengthening surveillance systems in this region are imperative.

### Recommendations

To prevent imported cases from negatively influencing the elimination goals, it is crucial to improve surveillance in the country’s borders [40]. It has demonstrated that Brazil’s surveillance system possesses data to conduct surveillance on imported malaria cases and subsequently implement control measures [38]. Enhancing health surveillance efforts in Brazil’s borders will facilitate the detection and timely treatment of infected individuals, thus preventing further transmission [10, 11].

Yet, the occurrence of imported cases along the country’s borders involves illegal gold miners deep in the Amazon forest and economic factors that extend beyond the reach of Brazil’s government. Addressing these issues requires coordinated efforts from various sectors of the Brazilian government, in partnership with other countries and international organizations. The complexity of this situation demands equally complex solutions [46].

Furthermore, practices in travel medicine [47, 48], such as counseling by health services [49], could be strengthened within the Brazilian National Health System. This would ensure that residents when travelling to endemic areas are informed about preventive measures and are aware of malaria symptoms upon returning to Brazil. To effectively educate travelers about the risks of malaria, health education initiatives should be reinforced and conducted by the surveillance teams [50]. In addition, presenting a negative malaria test upon return could prove beneficial [45]. This approach would enable the prompt detection and treatment of imported malaria cases, potentially reducing the incidence of severe cases [26, 51].

Similarly, as suggested for the Australian region, the establishment of a South America imported malaria surveillance network should be developed to enhance health surveillance efforts at the country’s borders [44].

### Limitations

The findings of this study offer new perspectives into malaria cases imported into Brazil from other countries, shedding light on the significant role of the African continent in this context. Moreover, it underscores the potential threat posed by these imported cases to Brazil’s elimination goals.

Key limitations of this study warrant acknowledgment are: The reliance on secondary data may have implications for data quality and representativeness particularly any malaria case data with missing country origin and non-reported cases.

Also, related to the data quality, the percentage of missing data poses a significant constraint for a more consistent analysis. For instance, over 34% of the imported cases notified in the Extra-Amazon area have unknown education levels (Table 1) and 29.6% of occupation information are “other” (unspecified). Therefore, it is inappropriate to assume that this proportion of missing data is equally distributed across all education and occupation levels. If those data be distributed in a non-random way, the profile of imported cases could change substantially. It is necessary for the state’s health departments and the BMoH to promote actions that better capacitate health surveillance teams, so that the epidemiological investigation of these cases can be improved, and inaccuracies can be reduced.

Challenges associated with accessing healthcare services in remote Amazonian areas may have led to an underestimation of the number of imported cases analyzed in this paper [52]. Nevertheless, it’s important to note that the information utilized in this study originates from established Information Systems and is representative at a national level [53].

Additionally, it is possible that the data analyzed represents only a minor part of real malaria cases, as imported cases are often associated with illegal gold mining in the Amazon [46, 54], so these "workers" may not seek diagnosis and treatment at official malaria centers. Unfortunately, this underreporting has not yet been quantified, so it is suggested that primary data collection studies try to quantify these underreport levels for a better comprehension of the reality of the malaria cases among gold miners.

## Conclusions

The study unveils a diminishing trend in imported malaria cases in Brazil, attributed to the Covid-19 pandemic and a broader reduction in malaria across the Americas Region. However, the substantial population migration from Latin America countries to Brazil presents a formidable challenge to the nation’s health surveillance efforts and could jeopardize its elimination goals. The present landscape underscores the imperative for enhanced cross-border collaboration to bolster surveillance, prevention, and control measures targeted at imported malaria cases.

## Supporting information

S1 Data Data repository. (CSV)

## References

[1] WHO. World malaria report 2023. Geneva: World Health Organization; 2023.

[2] Talapko J, Škrlec I, Alebić T, Jukić M, Včev A. Malaria: The past and the present. Vol. 7, Microorganisms. MDPI AG; 2019. 10.3390/microorganisms7060179 PMC6617065 31234443

[3] Fitzpatrick C, Engels D. Leaving no one behind: a neglected tropical disease indicator and tracers for the Sustainable Development Goals: Box 1. Int Health. 2016 Mar 3;8(suppl 1):i15–8. doi: 10.1093/inthealth/ihw002 26940304 PMC4777229

[4] Griffing SM, Tauil PL, Udhayakumar V, Silva-Flannery L. A historical perspective on malaria control in Brazil. Mem Inst Oswaldo Cruz. 2015 Sep 1;110(6):701–18. doi: 10.1590/0074-02760150041 26517649 PMC4667572

[5] Geleta G, Ketema T. Severe Malaria Associated with Plasmodium falciparum and P. vivax among Children in Pawe Hospital, Northwest Ethiopia. Malar Res Treat. 2016 Mar 7;2016:1–7. doi: 10.1155/2016/1240962 27047701 PMC4800101

[6] Ferreira MU, Castro MC. Malaria Situation in Latin America and the Caribbean: Residual and Resurgent Transmission and Challenges for Control and Elimination. In 2019. p. 57–70. doi: 10.1007/978-1-4939-9550-9_4 31267493

[7] Brazil. Epidemiological Report–Malaria 2021. Ministry of Health of Brazil. 2021.

[8] Oliveira-Ferreira J, Lacerda MV, Brasil P, Ladislau JL, Tauil PL, Daniel-Ribeiro CT. Malaria in Brazil: an overview [Internet]. Vol. 9, Malaria Journal. 2010. Available from: http://www.malariajournal.com/content/9/1/115 10.1186/1475-2875-9-115 PMC2891813 20433744

[9] Garcia KKS, Abrahão AA, Oliveira AFM, Henriques KMD, de Pina-Costa A, Siqueira AM, et al. Malaria time series in the extra-Amazon region of Brazil: epidemiological scenario and a two-year prediction model. Malar J. 2022;21(1). doi: 10.1186/s12936-022-04162-1 35641976 PMC9153870

[10] Arisco NJ, Peterka C, Castro MC. Cross-border malaria in Northern Brazil. Malar J. 2021 Dec 6;20(1):135. doi: 10.1186/s12936-021-03668-4 33676522 PMC7937307

[11] da Silva NS, da Silva-Nunes M, Malafronte RS, Menezes MJ, D’Arcadia RR, Komatsu NT, et al. Epidemiology and control of frontier malaria in Brazil: Lessons from community-based studies in rural Amazonia. Trans R Soc Trop Med Hyg. 2010 May;104(5):343–50. doi: 10.1016/j.trstmh.2009.12.010 20106494

[12] WHO. Disease Surveillance for Malaria Control: An Operational Manual. Geneva, Switzerland. 2012.

[13] WHO. Global technical strategy for malaria 2016–2030. Geneva, Switzerland. 2021. Available at: https://www.who.int/publications/i/item/9789240031357; 2015.

[14] Datasus. TABNET DATASUS. Avaiable at: https://datasus.saude.gov.br/informacoes-de-saude-tabnet/.

[15] R Core Team. (2023). R: A Language and Environment for Statistical Computing. Vienna ARF for SC. R Core Team. (2023).

[16] Prais SJ, Winsten CB. Trend estimators and serial correlation. Cowles Commission discussion paper Stat No. 383, Chicago, 1954.

[17] Bottomley C, Ooko M, Gasparrini A, Keogh R. In praise of Prais‐Winsten: An evaluation of methods used to account for autocorrelation in interrupted time series. Stat Med. 2023 Apr 15;42(8):1277–88. doi: 10.1002/sim.9669 36722328 PMC10946734

[18] Antunes JLF, Cardoso MRA. Using time series analysis in epidemiological studies Epidemiologia e Serviços de Saúde. 2015 Sep;24(3):565–76.

[19] Bottomley C, Scott JAG, Isham V. Analysing Interrupted Time Series with a Control. Epidemiol Methods. 2019 Dec 18;8(1).

[20] Lopez Bernal J, Cummins S, Gasparrini A. Interrupted time series regression for the evaluation of public health interventions: a tutorial. Int J Epidemiol. 2016 Jun 9;dyw098. 10.1093/ije/dyw098 PMC5407170 27283160

[21] Berra TZ, Ramos ACV, Alves YM, Tavares RBV, Tartaro AF, Nascimento MC do, et al. Impact of Covid-19 on Tuberculosis Indicators in Brazil: A Time Series and Spatial Analysis Study. Trop Med Infect Dis. 2022 Sep 14;7(9):247. doi: 10.3390/tropicalmed7090247 36136658 PMC9500936

[22] Ballard M, Olsen HE, Millear A, Yang J, Whidden C, Yembrick A, et al. Continuity of community-based healthcare provision during Covid-19: a multicountry interrupted time series analysis. BMJ Open. 2022 May;12(5):e052407. doi: 10.1136/bmjopen-2021-052407 35545397 PMC9096055

[23] QGIS. QGIS Geographic Information System. QGIS Association. Version 2.18. http://www.qgis.org.

[24] Bou-Karroum L, Khabsa J, Jabbour M, Hilal N, Haidar Z, Abi Khalil P, et al. Public health effects of travel-related policies on the Covid-19 pandemic: A mixed-methods systematic review. Journal of Infection. 2021 Oct;83(4):413–23. doi: 10.1016/j.jinf.2021.07.017 34314737 PMC8310423

[25] Pina-Costa A de, Brasil P, Santi SM Di, Araujo MP de, Suárez-Mutis MC, ACF e S, et al. Malaria in Brazil: what happens outside the Amazonian endemic region. Mem Inst Oswaldo Cruz. 2014 Aug;109(5):618–33. doi: 10.1590/0074-0276140228 25185003 PMC4156455

[26] Trampuz A, Jereb M, Muzlovic I, Prabhu RM. Clinical review: Severe malaria. Crit Care. 2003;7(4):315. doi: 10.1186/cc2183 12930555 PMC270697

[27] Paquet D, Jung L, Trawinski H, Wendt S, Lübbert C. Fever in the returning traveler. Dtsch Arztebl Int. 2022 Jun 7; doi: 10.3238/arztebl.m2022.0182 35469592 PMC9492913

[28] Brazil. Epidemiological Report–Malaria 2020. Ministry of Health of Brazil. 2020.

[29] Brazil. National Malaria Elimination Plan. 1st ed. Vol. 1. Ministry of Health of Brazil. 2022.

[30] Observatório das Migrações Internacionais. DataMigra BI panel [Online]. Available at: https://app.powerbi.com/view?r=eyJrIjoiNDFiODhmMjUtNmRiNy00MWMzLThjOTAtZTdlZGZjZmViZjg1IiwidCI6ImVjMzU5YmExLTYzMGItNGQyYi1iODMzLWM4ZTZkNDhmODA1OSJ9&pageName=ReportSection9b3637a54858b0741fea. Accessed at: May 18, 2024.

[31] Grillet ME, Hernández-Villena J V, Llewellyn MS, Paniz-Mondolfi AE, Tami A, Vincenti-Gonzalez MF, et al. Venezuela’s humanitarian crisis, resurgence of vector-borne diseases, and implications for spillover in the region. Lancet Infect Dis. 2019 May;19(5):e149–61. doi: 10.1016/S1473-3099(18)30757-6 30799251

[32] Wetzler EA, Marchesini P, Villegas L, Canavati S. Changing transmission dynamics among migrant, indigenous and mining populations in a malaria hotspot in Northern Brazil: 2016 to 2020. Malar J. 2022 Dec 19;21(1):127. doi: 10.1186/s12936-022-04141-6 35439994 PMC9018056

[33] de Aguiar Barros J, Granja F, Pequeno P, Marchesini P, Ferreira da Cruz M de F. Gold miners augment malaria transmission in indigenous territories of Roraima state, Brazil. Malar J. 2022 Nov 29;21(1):358. doi: 10.1186/s12936-022-04381-6 36447220 PMC9706895

[34] Bouma MJ, Dye C. Cycles of malaria associated with El Niño in Venezuela. JAMA. 1997 Dec 3;278(21):1772–4. 9388155

[35] Bouma MJ, Siraj AS, Rodo X, Pascual M. El Niño‐based malaria epidemic warning for Oromia, Ethiopia, from August 2016 to July 2017. Tropical Medicine & International Health. 2016 Nov 7;21(11):1481–8. 27580403 10.1111/tmi.12776

[36] Dhiman RC, Sarkar S. El Niño Southern Oscillation as an early warning tool for malaria outbreaks in India. Malar J. 2017 Dec 20;16(1):122. 28320394 10.1186/s12936-017-1779-y PMC5359847

[37] Laporta GZ, Grillet ME, Rodovalho SR, Massad E, Sallum MAM. Reaching the malaria elimination goal in Brazil: a spatial analysis and time-series study. Infect Dis Poverty. 2022 Dec 5;11(1):39. doi: 10.1186/s40249-022-00945-5 35382896 PMC8981179

[38] Arisco NJ, Peterka C, Castro MC. Imported malaria definition and minimum data for surveillance. Sci Rep. 2022 Oct 26;12(1):17982. doi: 10.1038/s41598-022-22590-6 36289250 PMC9605982

[39] Gomes M do SM, Menezes RA de O, Vieira JLF, Mendes AM, Silva G de V, Peiter PC, et al. Malaria in the borders between Brazil and French Guiana: social and environmental health determinants and their influence on the permanence of the disease. Saúde e Sociedade. 2020;29(2).

[40] Wangdi K, Wetzler E, Marchesini P, Villegas L, Canavati S. Cross-border malaria drivers and risk factors on the Brazil–Venezuela border between 2016 and 2018. Sci Rep. 2022 Apr 11;12(1):6058. doi: 10.1038/s41598-022-09819-0 35411064 PMC9001644

[41] Louzada J, de Almeida NCV, de Araujo JLP, Silva J, Carvalho TM, Escalante AA, et al. The impact of imported malaria by gold miners in Roraima: characterizing the spatial dynamics of autochthonous and imported malaria in an urban region of Boa Vista. Mem Inst Oswaldo Cruz. 2020;115. 10.1590/0074-02760200043 PMC7350773 32667459

[42] MacDonald AJ, Mordecai EA. Amazon deforestation drives malaria transmission, and malaria burden reduces forest clearing. Proc Natl Acad Sci USA. 2019;116(44):22212–22218. doi: 10.1073/pnas.1905315116 31611369 PMC6825316

[43] Vittor AY, Laporta GZ, Sallum MAM, Walker RT. The COVID-19 crisis and Amazonia’s indigenous people: Implications for conservation and global health. World Dev. 2021;145:105533. doi: 10.1016/j.worlddev.2021.105533 36570383 PMC9758534

[44] Sohail A, Barry A, Auburn S, Cheng Q, Lau CL, Lee R, et al. Imported malaria into Australia: surveillance insights and opportunities. J Travel Med. 2024 Apr 6;31(3). doi: 10.1093/jtm/taad164 38127641 PMC10998534

[45] Zhu Y, Restrepo AC, Wang HB, Mills DJ, Liang RR, Liu ZB, et al. Malaria cases in China acquired through international travel, 2013–2022. J Travel Med. 2024 Apr 9; 10.1093/jtm/taae056 PMC11646087 38591791

[46] Amaral PST, Garcia KKS, Suárez-Mutis MC, et al. Malaria in areas under mining activity in the Amazon: A review. Rev Soc Bras Med Trop. 2024;57:e002002024. Published 2024 Jun 24. doi: 10.1590/0037-8682-0551-2023 38922216 PMC11210384

[47] Pavli A, Lymperi I, Katerelos P, Maltezou HC. Knowledge and practice of malaria prophylaxis among travel medicine consultants in Greece. Travel Med Infect Dis. 2012 Sep;10(5–6):224–9. doi: 10.1016/j.tmaid.2012.09.006 23142310

[48] Peetermans WE, Van Wijngaerden E. Implementation of pretravel advice: good for malaria, bad for diarrhea. Acta Clin Belg. 2001 Oct 9;56(5):284–8. 11770223 10.1179/acb.2001.042

[49] Chaves T do SS, Monteiro WM, Alves JR, Lacerda M, Lopes MH. Pre-travel malaria chemoprophylaxis counselling in a public travel medicine clinic in São Paulo, Brazil. Malar J. 2017 Dec 7;16(1):64. doi: 10.1186/s12936-017-1713-3 28173862 PMC5297158

[50] Genton B, D’Acremont V. Malaria Prevention in Travelers. Infect Dis Clin North Am. 2012 Sep;26(3):637–54. doi: 10.1016/j.idc.2012.05.003 22963775

[51] Marasinghe DH, Cheaveau J, Meatherall B, Kuhn S, Vaughan S, Zimmer R, et al. Risk of malaria associated with travel to malaria-endemic areas to visit friends and relatives: a population-based case–control study. CMAJ Open. 2020 Jan;8(1):E60–8. doi: 10.9778/cmajo.20190070 31992561 PMC6996033

[52] Palma-Cuero M, Machado MB, Graça JTB, Anjos NB dos, Pereira RS, Suárez-Mutis MC. Malaria at international borders: challenges for elimination on the remote Brazil-Peru border. Rev Inst Med Trop Sao Paulo. 2022;64. doi: 10.1590/S1678-9946202264029 35384960 PMC8993150

[53] Coelho Neto GC, Chioro A. After all, how many nationwide Health Information Systems are there in Brazil? Cad Saude Publica. 2021;37(7). 10.1590/0102-311X00182119 34287586

[54] Martins-Filho PR, Araújo FWC, Santos-Júnior LC, et al. The increase in cases and deaths from malaria in the Brazilian Yanomami territory is associated with the spread of illegal gold mining in the region: A 20-year ecological study. Travel Med Infect Dis. 2024;57:102686. doi: 10.1016/j.tmaid.2023.102686 38159876

